# Fecal miR‐223 is a noninvasive biomarker for estimating Crohn's disease activity

**DOI:** 10.1002/iid3.1131

**Published:** 2023-12-28

**Authors:** Juanjuan Zhang, Zhen Guo, Zhiming Wang, Weiming Zhu, Qiurong Li

**Affiliations:** ^1^ Research Institute of General Surgery Jinling Hospital Nanjing China

**Keywords:** Crohn's disease, disease activity, fecal biomarker, microRNA‐223

## Abstract

**Introduction:**

MicroRNA‐223 (miR‐223) has emerged as a promising noninvasive biomarker for Crohn's disease (CD). However, it is unclear which tissue derived miRNA‐223 can more accurately estimate CD disease activity.

**Materials and Methods:**

To collect serum, terminal ileal mucosa biopsy and fecal samples from CD patients and healthy controls. The CD Activity Index (CDAI) score, Montreal classification, maintenance medicines, peripheral blood inflammatory markers, fecal calprotectin (FC) and the Simple Endoscopic Score for CD (SES‐CD) were recorded. To compare the expression of miR‐223 in the serum, intestinal tissue, and feces.

**Results:**

MiR‐223 expression levels in the serum, intestinal tissue and feces of CD patients were significantly higher than those of controls. The level of miR‐223 in the serum, intestinal tissue and feces increased significantly in active CD patients compared with that in inactive CD patients. The levels of serum, intestinal tissue and fecal miR‐223 were correlated with the CDAI. Serum miR‐223 was also correlated with C‐reactive protein (CRP) and IL‐6, tissue miR‐223 correlated with IL‐6 and FC, and fecal miR‐223 correlated with FC. In terms of the association with FC, fecal miR‐223 had a higher Spearman r value than tissue miR‐223. The area under the curve (AUC) values of serum, tissue and fecal miR‐223 to diagnose CD were similar to those of CRP and FC (AUC > 0.8). The AUC values of tissue and fecal miR‐223 to evaluate CD disease activity were 0.832 and 0.818, respectively, and were higher than serum miR‐223, CRP and FC. Fecal miR‐223 had a higher specificity of 92.3%.

**Conclusions:**

Fecal miR‐223 might be a novel, noninvasive biomarker for estimating the disease activity of CD patients.

## 1 INTRODUCTION

Inflammatory bowel disease (IBD) is chronic inflammation of the gastrointestinal tract, including Crohn's disease (CD) and ulcerative colitis (UC). CD is manifested as patchy transmural inflammation that impact any part of the gastrointestinal tract, whereas UC has inflammation typically limited to the colon or rectum. CD is diagnosis is based on medical history, clinical manifestations and assessments including laboratory tests and endoscopic and radiological images. Endoscopy plays a key role in the diagnosis and assessment of CD. However, it is invasive, costly, burdensome to patients, and carries the risk of serious complications.^1^ The most common biomarkers for evaluating disease activity and monitoring inflammation during treatment for CD are C‐reactive protein (CRP) and fecal calprotectin (FC). IL‐6 is also an inflammatory marker involved the pathogenesis of CD and used to evaluate inflammatory response. However, these biomarkers are not specific to CD and are elevated in many other diseases.^2^, ^3^, ^4^, ^5^, ^6^ Therefore, a novel, noninvasive and applicable biomarker is needed for clinics to optimize the diagnosis and assessment of CD.

MicroRNAs (MiRNAs) are small, single‐stranded noncoding RNAs that have been found to be involved in the initiation, development and progression of CD, and they may have the potential to be used as biomarkers and therapeutic targets.^7^ MiRNAs have been identified in serum, other body fluids and excrements including stool, bile, saliva, and urine.^8^, ^9^ MiR‐223 is a key modulator for regulating the differentiation and activation of myeloid cells, especially neutrophils and macrophages.^10^ Recently, it has been demonstrated that the expression of miR‐223 is abnormal in CD.^11^, ^12^, ^13^, ^14^ In this study, we compared the expression of miR‐223 in the serum, tissue and feces of CD patients to determine whether miR‐223 is a more precise and noninvasive biomarker for CD.

## 2 MATERIALS AND METHODS

### 2.1 Material

This was a prospective study that enrolled 24 CD patients and 6 healthy controls at Jinling Hospital between January and March 2020. The inclusion criteria involved patients with CD diagnosed by clinical, endoscopic, and histological criteria and healthy controls with matching body mass indexes as well as no history of chronic diseases.^15^ The exclusion criteria included patients with a history of intestinal resection, intestinal tuberculosis, cancer, heart failure, hepatic disorders or renal disorders. All participants were provided complete information about the study. This study was approved by the Institutional Ethics Committee of Jinling Hospital (2021NZKY‐010‐1).

### 2.2 Methods

The clinical disease activity was graded according to the CD Activity Index (CDAI) score. Patients were divided into two groups according to disease activity. The active group (*n* = 11) included patients with active disease (CDAI > 150), and the inactive group (*n* = 13) included patients in remission (CDAI ≤ 150). Endoscopies were performed and assessed by the Simple Endoscopic Score for CD (SES‐CD). The site of disease was defined according to the Montreal classification.^16^

Blood samples were taken after admission, and serum samples were isolated by centrifugation from 3 mL of total blood. Stool samples were collected before bowel preparation (preferably from the first stool in the morning). All patients and healthy controls underwent ileo‐colonoscopies. For CD patients, three punch biopsies were obtained from lesions of terminal ileal mucosa, and for healthy controls from normal terminal ileal mucosa during the endoscopies. Serum, feces and tissue samples were stored at −80℃ until miR‐223 analysis.

Serum miRNA was isolated using the QIAGEN PAXgene Blood miRNA Kit (QIAGEN, Chatsworth), biopsy miRNA was isolated using the miRcute miRNA Isolation Kit (Tiange) and fecal miRNA was isolated using the QIAGEN RNeasy Mini Kit (QIAGEN). The isolation procedure of miRNA followed the manufacturer's recommendations. Total RNA was reverse‐transcribed, and quantitative real‐time polymerase chain reaction (PCR) analyses were performed on an ABI Step One Plus Real‐time PCR System.

### 2.3 Statistical analysis

Data were analyzed with SPSS 17.0. Continuous variables are expressed as the mean ± SD, and noncontinuous variables are expressed as the frequency and percentage. Experimental data analyses between two groups were performed with unpaired, two‐tailed Student's t‐tests or the χ^2^ test. One‐way analysis of variance was used for comparing all pairs of groups. The association between two variables was assessed by the Spearman rank correlation coefficient (r) for nonparametric correlations. Significant differences were determined at *p* < .05. Receiver operating characteristic (ROC) curve analysis was performed to diagnose CD and evaluate disease activity defined by the area under the curve (AUC).

## 3 RESULTS

**Figure 1:**
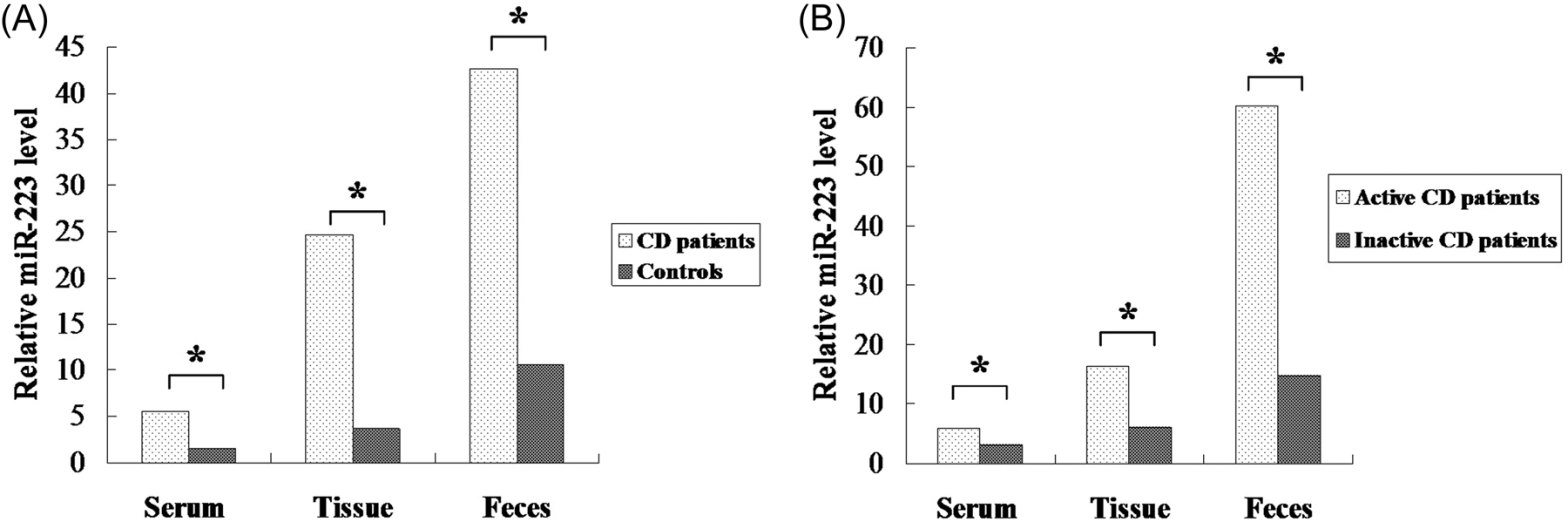
MiR‐223 expression levels in the serum, tissue and feces (A) The level of miR‐223 in the serum, tissue, and feces between CD patients and controls. (B) The level of miR‐223 in the serum, tissue, and feces between inactive CD and active patients, where * indicates *p* < .05.

**Figure 2:**
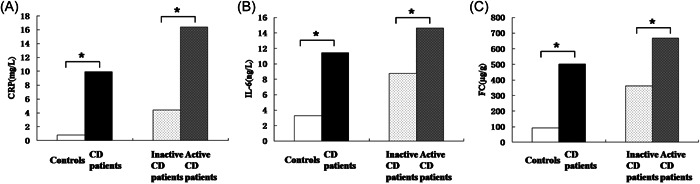
The level of CRP, IL‐6 and FC between two groups (A) The level of CRP between controls and CD patients as well as active patients and inactive patients. (B) The level of IL‐6 between controls and CD patients as well as active patients and inactive patients. (C) The level of FC between controls and CD patients as well as active patients and inactive patients, where * indicates *p* < .05.

The clinical characteristics of 24 patients with CD and 6 healthy controls are shown in Table 1. Compared with the controls, the miR‐223 expression of CD patients was significantly higher, with an increase of 2.9‐fold in serum, 6.1‐fold in tissue and 7.0‐fold in feces (Figure 1A). Based on the CDAI, there were 13 CD patients in the inactive group and 11 CD patients in the active group. The miR‐223 expression in the serum, tissue and feces was significantly increased in patients with active disease, with an increase of 1.9‐fold in serum, 2.7‐fold in tissue and 4.1‐fold in feces (Figure 1B). Comparing CD patients with controls, as well as inactive and active CD patients, CRP, IL‐6 and FC had significant differences (Figure 2).

**Table 1:** Clinical characteristics of CD patients.

	Controls (*n* = 6)	CD patients (*n* = 24)
Age (years)	43.5 ± 12.52	37.46 ± 11.96
Male (%)	4 (66.67%)	19 (79.17%)
Disease duration (years)		8.58 ± 3.97
Montreal Classification		
Age (%)		
A1 (≤16 years)	0 (0%)	0 (0%)
A2 (17–40 years)	5 (83.33%)	17 (70.83%)
A3 (＞40 years)	1 (16.67%)	7 (29.17%)
Disease location (%)		
L1 (ileal)		3 (12.5%)
L2 (colonic)		
L3 (ileocolonic)		18 (75%)
L1 + L4 (upper gastrointestinal tract)		1 (4.17%)
L3 + L4		2 (8.33%)
Disease behavior (%)		
B1 (inflammatory)		4 (16.67%)
B2 (stricturing)		9 (37.5%)
B3 (penetrating)		1 (4.17%)
B2 + 3		10 (41.67%)
+ P (perianal)		5 (20.83%)
Medication		
No		3 (12.5%)
Mesalazine		1 (4.17%)
Thalidomide＋EN		2 (8.33%)
Azathioprim＋EN		2 (8.33%)
Sulfasalazine		1 (4.17%)
Sulfasalazine＋EN		1 (4.17%)
EN		11 (45.83%)
TPN		2 (8.33%)
Infliximab		1 (4.17%)

Abbreviations: EN, enteral nutrition; TPN, total parenteral nutrition.

**Figure 3:**
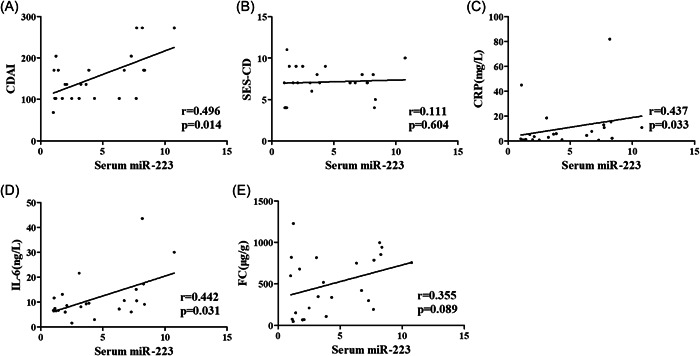
Correlation of serum miR‐223 with CDAI, SES‐CD, CRP, IL‐6 and FC in CD patients (A) Correlation analysis of serum miR‐223 with CDAI in CD patients. (B) Correlation analysis of serum miR‐223 with SES‐CD in CD patients. (C) Correlation analysis of serum miR‐223 with CRP in CD patients. (D) Correlation analysis of serum miR‐223 with IL‐6 in CD patients. (E) Correlation analysis of serum miR‐223 with FC in CD patients.

**Figure 4:**
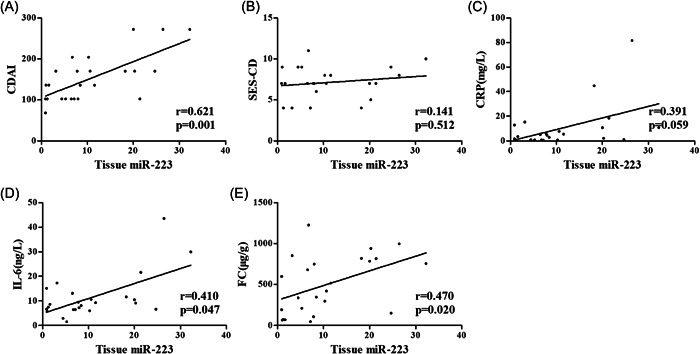
Correlation of tissue miR‐223 with CDAI, SES‐CD, CRP, IL‐6 and FC in CD patients. (A) Correlation analysis of tissue miR‐223 with CDAI in CD patients. (B) Correlation analysis of tissue miR‐223 with SES‐CD in CD patients. (C) Correlation analysis of tissue miR‐223 with CRP in CD patients. (D) Correlation analysis of tissue miR‐223 with IL‐6 in CD patients. (E) Correlation analysis of tissue miR‐223 with FC in CD patients.

**Figure 5:**
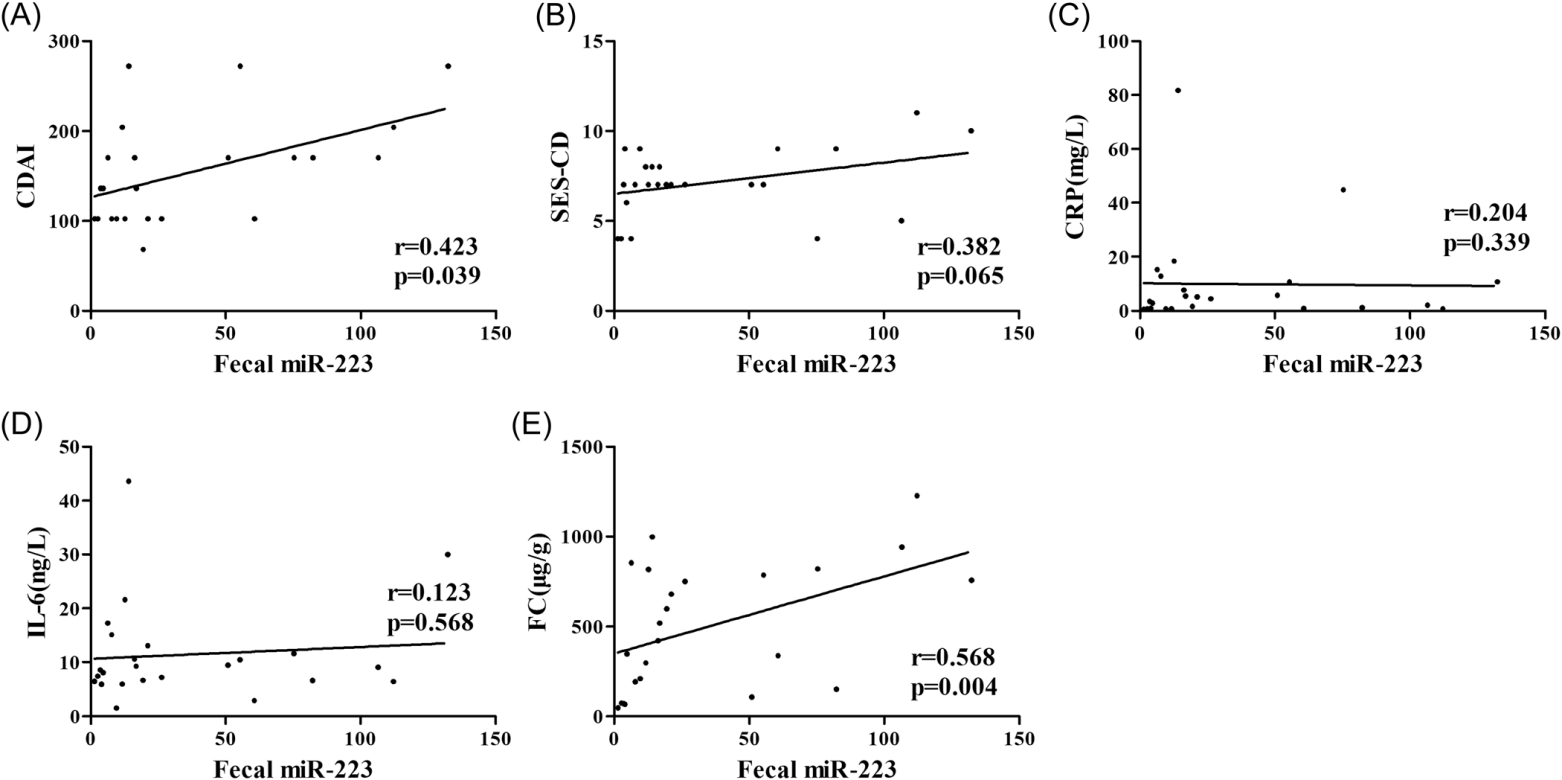
Correlation of fecal miR‐223 with CDAI, SES‐CD, CRP, IL‐6 and FC in CD patients. (A) Correlation analysis of fecal miR‐223 with CDAI in CD patients. (B) Correlation analysis of fecal miR‐223 with SES‐CD in CD patients. (C) Correlation analysis of fecal miR‐223 with CRP in CD patients. (D) Correlation analysis of fecal miR‐223 with IL‐6 in CD patients. (E) Correlation analysis of fecal miR‐223 with FC in CD patients.

The correlations of serum, tissue and fecal miR‐223 with CDAI, SES‐CD, CRP, IL‐6, and FC in CD patients were analyzed to identify whether the miR‐223 expression level had potential diagnostic value (Figures 3, 4 and 5). Serum miR‐223 was significantly correlated with CDAI (*r* = .496, *p* = .014), CRP (*r* = .437, *p* = .033) and IL‐6 (*r* = .442, *p* = .031). Tissue miR‐223 was significantly correlated with CDAI (*r* = .621, *p* = .001), IL‐6 (*r* = .410, *p* = .047) and FC (*r* = .470, *p* = .020). Fecal miR‐223 was significantly correlated with CDAI (r = .423, *p* = .039) and FC (*r* = .568, *p* = .004).

**Figure 6:**
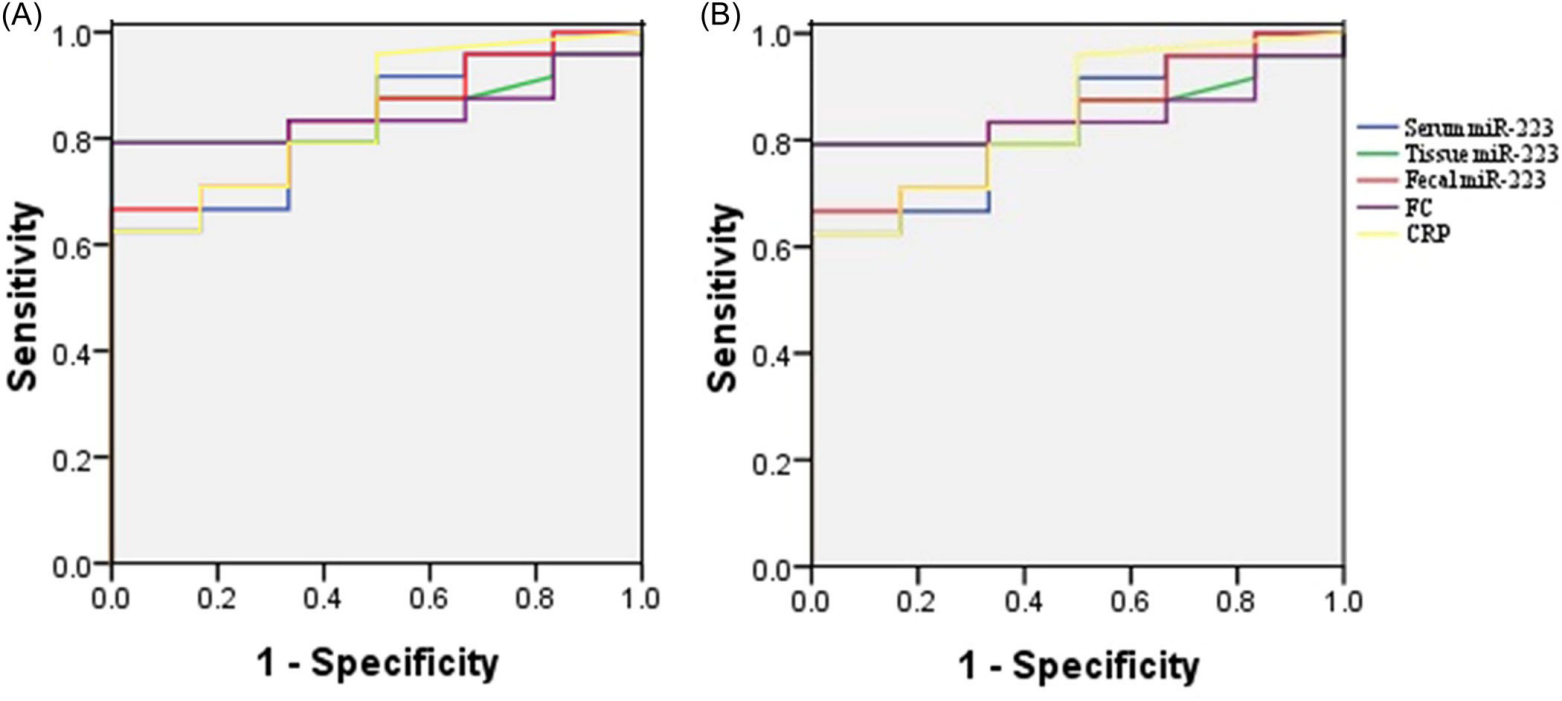
ROC for miR‐223 in serum, tissue and feces to diagnose CD and evaluate disease activity. (A) ROC for miR‐223 in the serum, tissue and feces to diagnose CD. (B) ROC for miR‐223 in the serum, tissue, and feces to evaluate CD disease activity.

The ROC analysis of CD patients versus controls revealed that the AUC values for miR‐223 in serum, tissue, and feces, as well as for CRP and FC were 0.826, 0.851, 0.840, 0.844, and 0.847, respectively (Figure 6A). The ROC analysis of active patients versus inactive patients revealed that the AUC values for miR‐223 in serum, tissue and feces, as well as for CRP and FC were 0.741, 0.832, 0.818, 0.671, and 0.776, respectively (Figure 6B). The AUC of tissue miR‐223 and fecal miR‐223 were the highest among these markers; tissue miR‐223 had a sensitivity of 72.7% and a specificity of 84.6% (cutoff: 9.36), and fecal miR‐223 had a sensitivity of 63.6% and a specificity of 92.3% (cutoff: 38.61).

## 4 DISCUSSION

This study showed that the expression of miR‐223 in the serum, tissue and feces of CD patients was significantly increased compared to that in healthy controls, and the miR‐223 expression in the serum, tissue and feces of active CD patients was higher than that in inactive CD patients. Moreover, the expression of serum miR‐223, tissue miR‐223 and fecal miR‐223 correlated with the CDAI. Serum miR‐223 also correlated with CRP and IL‐6, and tissue miR‐223 and fecal miR‐223 correlated with FC. The AUC values of miR‐223 in serum, tissue and feces to diagnose CD were all greater than 0.8, which were similar to those of CRP and FC; the AUC values of tissue and fecal miR‐223 for evaluating CD disease activity were 0.832 and 0.818, respectively, which were more precise than those of serum miR‐223, CRP and FC. Fecal miR‐223 had a higher specificity than tissue miR‐223.

To identify a precise and noninvasive biomarker for CD was the aim of this study. CD is a major type of IBD with the terminal ileum most commonly involved. CRP and FC are the most common noninvasive biomarkers for diagnosing CD and monitoring disease activity. CRP is an acute phase reactant that is elevated in multiple conditions and systemic inflammatory states, but it is not a specific biomarker for CD.^6^ FC is a protein produced by neutrophils, monocytes and macrophages and then released into feces after the degranulation of these cells, and used to evaluate intestinal inflammation.^2^ It has been reported that FC can be used to diagnose CD, predict relapse, and assess the response to therapy.^17^, ^18^ But FC is lack of specificity, because FC is not only used for clinical diagnosis of CD but also is increased in many other intestinal disease such as infectious colitis, irritable bowel syndrome and colorectal cancer.^2^, ^3^, ^4^, ^5^, ^6^ Thus, a novel noninvasive biomarker of CD is needed in clinical practice.

MiRNAs were found to be new biomarkers of many diseases, including inflammatory diseases and cancer,^5^, ^7^, ^19^ and fecal miRNAs are potential biomarkers of colon cancer and pancreatic cancer.^20^, ^21^ MiR‐223 is highly expressed in myeloid cells especially neutrophils, and regulate neutrophils function in inflammatory diseases.^10^ MiR‐223 is involved in the development of IBD, including NF‐κB signaling, the IL23/Th17 pathway and the NLRP3–IL‐1β circuit, and could be a biomarker and therapeutic target.^22^, ^23^, ^24^ In our previous study, we demonstrated that miR‐223 expression in the terminal ileum and colon was upregulated in the in dextran sodium sulfate induced colitis, and upregulation of miR‐223 could attenuate the clinical signs of experimental colitis and colonic inflammation, which was likely mediated by inhibiting the production of pro‐inflammatory cytokines via the IL‐6/STAT3 signaling pathway.^25^ Furthermore, it is reported that miR‐223 in CD patients is increased in blood, intestinal tissues and feces. Wu et al. found that the expression levels of six miRNAs including miR‐223 were increased in CD terminal ileal.^26^ Wang et al. reported that expression of serum miR‐223 is increased in patients with CD and correlated with disease activity.^12^ Schönauen et al. also found that the serum and fecal miR‐223 expression in CD patients were higher.^27^ However, it is unclear which tissue derived miRNA‐223 can more accurately estimate CD disease activity.

Serum miR‐223 correlated with CRP and IL‐6. Tissue miR‐223 and fecal miR‐223 correlated with FC, and we hypothesized that tissue miR‐223 and fecal miR‐223 could reflect intestinal inflammation. Fecal miR‐223 expression was higher than that in serum and tissue in comparing CD patients and healthy controls and in comparing active CD patients and inactive CD patients, and fecal miR‐223 correlated with the CDAI (*r* = .423, *p* = .039). Combined with the results that fecal miR‐223 strongly correlated with FC compared with tissue miR‐223, fecal miR‐223 could be a noninvasive biomarker to replace FC in the evaluation of intestinal inflammation. MiRNAs have been identified in gut epithelial cells and +4 niche‐derived Hopx‐expressing cells as two main sources of fecal miRNAs, and fecal miRNAs are a normal component of the gut lumen.^28^ Therefore, fecal miRNA expression could more accurately evaluate intestinal inflammation or mucosal healing in gastrointestinal diseases. Schönauen et al., demonstrated that miRNAs in feces correlate with disease activity and may be considered a potential tool for further biomarker research in CD.^27^ In addition, the AUC value of fecal miR‐223 to evaluate CD disease activity was greater than that of serum miR‐223, CRP and FC with a higher specificity of 92.3%. These data suggest that fecal miR‐223 could be used a noninvasive biomarker to assess intestinal inflammation and estimate CD disease activity.

However, this study had some limitations. First, the samples size was small, and it is necessary to include more samples for analyze in the future. Second, the study population involved only individuals of Chinese decent.

In conclusion, the expression of miR‐223 in the serum, tissue and feces of CD patients was significantly increased compared to that in healthy controls, and the miR223 expression in serum, tissue and feces of active CD patients was higher than that in inactive CD patients. Fecal miR‐223 could be used to assess disease activity with a higher accuracy and specificity than CRP, FC, serum and tissue miR‐223. Therefore, fecal miR‐223 could be a noninvasive biomarker to estimate intestinal inflammation as well as CD disease activity and would be applicable for clinical practice.

## AUTHOR CONTRIBUTIONS

**Juanjuan Zhang**: Conceptualization; data curation; formal analysis; funding acquisition; investigation; methodology; project administration; writing—original draft. **Zhen Guo**: Conceptualization; methodology; project administration. **Zhiming Wang**: Formal analysis; project administration; writing—original draft. **Weiming Zhu**: Conceptualization; project administration; writing—review and editing. **Qiurong Li**: Conceptualization; project administration; writing—review and editing.

## CONFLICT OF INTEREST STATEMENT

The authors declare no conflict of interest.

## Data Availability

The data that support the findings of this study are available from the corresponding author upon reasonable request.
